# ^32^P_i_ Labeled Transgenic Wheat Shows the Accumulation of Phosphatidylinositol 4,5-bisphosphate and Phosphatidic Acid Under Heat and Osmotic Stress

**DOI:** 10.3389/fpls.2022.881188

**Published:** 2022-06-14

**Authors:** Nazish Annum, Moddassir Ahmed, Khadija Imtiaz, Shahid Mansoor, Mark Tester, Nasir A. Saeed

**Affiliations:** ^1^Wheat Biotechnology Lab, Agricultural Biotechnology Division, National Institute for Biotechnology and Genetic Engineering, Constituent College Pakistan Institute of Engineering and Applied Sciences, Faisalabad, Pakistan; ^2^Center for Desert Agriculture, King Abdullah University of Science and Technology, Thuwal, Saudi Arabia

**Keywords:** heat stress, osmotic stress, PA, PIP_2_, ^32^P_i_, wheat

## Abstract

The ensuing heat stress drastically affects wheat plant growth and development, consequently compromising its grain yield. There are many thermoregulatory processes/mechanisms mediated by ion channels, lipids, and lipid-modifying enzymes that occur in the plasma membrane and the chloroplast. With the onset of abiotic or biotic stresses, phosphoinositide-specific phospholipase C (PI-PLC), as a signaling enzyme, hydrolyzes phosphatidylinositol 4,5-bisphosphate (PIP_2_) to generate inositol 1,4,5-trisphosphate (IP_3_) and diacylglycerol (DAG) which is further phosphorylated into phosphatidic acid (PA) as a secondary messenger and is involved in multiple processes. In the current study, a phospholipase C (PLC) signaling pathway was investigated in spring wheat (*Triticum aestivum* L.) and evaluated its four *AtPLC5* overexpressed (OE)/transgenic lines under heat and osmotic stresses through ^32^P_i_ radioactive labeling. Naturally, the wheat harbors only a small amount of PIP_2_. However, with the sudden increase in temperature (40°C), PIP_2_ levels start to rise within 7.5 min in a time-dependent manner in wild-type (*Wt*) wheat. While the Phosphatidic acid (PA) level also elevated up to 1.6-fold upon exposing wild-type wheat to heat stress (40°C). However, at the anthesis stage, a significant increase of ∼4.5-folds in PIP_2_ level was observed within 30 min at 40°C in *AtPLC5* over-expressed wheat lines. Significant differences in PIP_2_ level were observed in *Wt* and *AtPLC5-OE* lines when treated with 1200 mM sorbitol solution. It is assumed that the phenomenon might be a result of the activation of PLC/DGK pathways. Together, these results indicate that heat stress and osmotic stress activate several lipid responses in wild-type and transgenic wheat and can explain heat and osmotic stress tolerance in the wheat plant.

## Introduction

Sustainability in agriculture depends on growing suitable crops for a particular climate in the defined areas. Prolonged exposure to high temperatures drastically affects crop productivity. Elevated temperatures also result in osmotic stress from the water evaporation within the soil causing excessive salt accumulation. Heat, drought, and salt are the major abiotic stresses affecting crop yield. These stresses in combination are becoming quite common in heat and drought-hit areas. Among cereals, wheat is domesticated first and considered a major staple food crop globally (Tack et al., 2015; Abhinandan et al., 2018). Heat negatively affects the wheat grain yield. It is estimated that every 1°C rise in temperature results in 6% yield losses in wheat crops; however, it depends on the specific growth stage of the crop, time, duration, and intensity. An increase in temperature above the optimum value before and during anthesis results in embryo abortion in developing seeds, reducing the grain number/ear without affecting the grain weight, whereas, after anthesis, the onset of high temperature does not affect the number of grains per ear but reduce the grain size and weight by hampering grain filling ultimately affecting the crop yield (Foulkes et al., 2002; Weldearegay et al., 2012; Schmidt et al., 2020).

Plants are sessile eukaryotes and are very sensitive to even slight changes in their environment. There are some receptors present on the plant cell membrane that perceive stress (abiotic/biotic) signals and transduce this information downstream for the activation of certain stress-responsive genes. The ultimate product of coordinated action of these genes results in signal transcription/proteins synthesis, protein modification like ubiquitination, glycosylation, methylation, adaptors attachment, and subsequently scaffolding of the plants to adapt/survive under harsh environmental conditions (Trewavas and Malho, 1997; McCarty and Chory, 2000; Gilroy and Trewavas, 2001; Mahajan and Tuteja, 2005; Tuteja, 2007; Tuteja and Sopory, 2008).

Under extreme temperatures, plants tend to maintain their membrane integrity and fluidity, acting as a permeable barrier. According to a rough estimate, the membrane surface of a plant cell is recycled every 90–120 min (Munnik et al., 2021). These lipids have amphipathic properties and can be differentiated as sphingolipids, glycerolipids, and sterols based on their unique chemical structure and biophysical properties (Enrique Gomez et al., 2017). Among glycerolipids, phospholipids are predominantly present in the mitochondrial envelope and plasma membrane (PM), which play a vital role in the development of the plant, regulating their responses against particular environmental stimuli (Dubots et al., 2012; Niu and Xiang, 2018; Wang X. et al., 2020). Plant phospholipases are involved in the hydrolysis of phospholipids and can be divided into four categories, that is, *PLA 1* (phospholipase A1), *PLA 2* (phospholipase A2), *PLC* (phospholipase C), and *PLD* (phospholipase D). Within each category, there are subfamilies with different structures, substrates, and binding sites (Wang X. et al., 2020). Three types of PLCs are reported based on their cellular function and substrates specificity: (1) PI-PLC (Phosphatidylinositol-specific PLCs) hydrolyzes phosphoinositide (PPI); (2) *PC-PLC/NPC* (phosphatidylcholine-specific *PLC*/Non-specific phospholipase C) hydrolyzes the commonly present phospholipids like PC and PE; and (3) *GPI-PLC* (Glycosyl phosphatidylinositol PLC) hydrolyzes the proteins attached to the glycosylphosphatidylinositol (GPI) (Hong et al., 2016).

Extracellular signals activate the *PLC*s responsible for the production of inositol 1,4,5 trisphosphate (InsP3) and diacylglycerol (DAG). InsP3 travels to the cytoplasm to bind and activate the ligand-gated calcium channel also known as the InsP3 receptor to release Ca^+2^ from intracellular channels, whereas, DAG deals with the protein kinase C (PKC) family which has a C1 conserved domain. Massive intracellular processes due to increase or decrease in calcium and phosphorylation levels result in the activation and deactivation of various target proteins to respond against extracellular changes (Shiva et al., 2020; Hayes et al., 2021).

The signaling pathway of plant PI-PLC is somewhat different from mammals, for example, in plants, inositol 1, 4, and 5 trisphosphates (InsP3) could phosphorylate further to inositol hexakisphosphate (InsP6), which is responsible for the release of calcium ions from intracellular calcium reserves and similarly, phosphatidic acid (PA) which is a product of diacylglycerol (DAG) might act as a second messenger in this pathway (Munnik, 2014). PIP_2_ is presumably a substrate of PLC, hardly found in the plasma membrane of flowering plants (Simon et al., 2014; Zhang et al., 2018c). PLC hydrolyze PIP (Phosphatidylinositol 4 monophosphate), that is, also known as the precursor of PLC and can be found in abundance in the plasma membrane, but to date, the typical precursor of PLC in plants is unknown in *in vivo* analysis (Munnik, 2014). Likewise, DGPP (Diacylglycerol pyrophosphate) can function as an attenuator of PA signaling and as a generator of new signals, but it needs to be investigated further (van Schooten et al., 2006).

Plants tend to upregulate many *PLC* genes upon the onset of various biotic and abiotic stresses. In *Arabidopsis thaliana*, 9 *PI-PLCs* and 6 *NPC*s genes (Munnik, 2014), 4 *PI-PLC* and 5 *NPCs* in *Oryza sativa* (Rice) (Singh et al., 2013), 12 *PI-PLCs* and 9 *NPC*s genes in *Gossypium* spp. (Cotton) (Zhang et al., 2018a), 5 *PI-PLC* and 4 *NPC*s genes in *Zea mays* L. (Maize), while 12 *PLC* genes in *Glycine max* (Soybean) (Wang F. et al., 2015) are reported. An increase in PIP_2_ and PA had been observed in response to heat, salt, cold, drought, and ABA stresses (Alcázar-Román and Wente, 2008; Darwish et al., 2009; Mishkind et al., 2009; Arisz et al., 2013; Balogh et al., 2013; Simon et al., 2014; Zhang et al., 2018c). Earlier it is reported that *PLC* is involved in plant growth and development, for example, *PLC1* is known to contribute to pollen tube growth in tobacco and petunia (Dowd et al., 2006; Helling et al., 2006), over-expression of the *PLC2* gene can increase drought tolerance and regulate phytochrome level in *Brassica napus* (Das et al., 2005; Nokhrina et al., 2014), *PLC3* and *PLC9* contributing in generating thermotolerance in *Arabidopsis thaliana* (Zheng et al., 2012; Gao et al., 2014), upregulation of *AtPLC5* in response to drought stress could lead to subsequent novel phenotype including stunted root hair growth, reduced lateral root development, stomatal closure, and inhibition/reduction of seed germination (Zhang et al., 2018b,c). These findings are inconsistent with previous studies as reported on maize, tomato, and potato (Apone et al., 2003; Apostolakos et al., 2008; Wang et al., 2008; Vossen et al., 2010).

PI-PLC was initially reported in wheat in root plasma membrane vesicles (Melin et al., 1992). Based on their subfamily, genomic homology, and chromosomal position, a total of 26 *TaPLC* genes including 7 *NPC* genes have been reported in *Triticum aestivum* (wheat) which are located unevenly on 14 chromosomes (Wang X. et al., 2020), but to date, *TaPLC1* (Zhang et al., 2014; Wang X. et al., 2021) and *TaPI-PLC1-2B* have been cloned and investigated for salt, drought, heat, and cold stress (Khalil et al., 2011; Wang X. et al., 2020). However, *TaPLC5* has yet to be reported in the already identified wheat PLCs. There is growing evidence that phosphoinositide signaling is a major element of stress responses. It proposes that changes in the lipid signal levels are one of the early consequences of abiotic stresses. Therefore, this study focuses on investigating signaling phospholipids levels in response to high temperature and osmotic stresses. We observed that *AtPLC5* over-expression causes a dramatic increase in PIP_2_ and PA levels at tillering and anthesis stages. These are the crucial stages for wheat grain development at various duration in varying intensity levels of heat and osmotic stresses.

## Materials and Methods

### Plant Material

Seeds of local wheat cultivar Faisalabad-2008 was used as wild-type (*Wt*) and four transgenic wheat lines over-expressing (OE) *AtPLC5* gene were used in the current study. The transgenic lines *OE1* and *OE2* were processed under *CaMV35S* promoter, while lines *OE3* and *OE4* contained *UBQ10* promotor. These transgenic wheat lines were obtained through *Agrobacterium*-mediated plant transformation method using immature embryos as explant (Ishida et al., 2015), and putative transgenic wheat lines were screened out based on PCR, quantitative PCR, and antibiotic leaf dip assay. Nevertheless, morpho-physiologically best representative lines were selected and used in this study (unpublished data). Plants were grown in small pots containing peat moss in a greenhouse with a 16/8 h day length regime at 20°C. Leaf samples from transgenic wheat lines were collected from the greenhouse and processed for further experimentation.

### RNA Extraction and Q-PCR

The expression level of *AtPLC5* (At5g58690) transgene in wheat was measured using primer pairs: 5’GT CGCTTTCAACATGCAGGG3’ and 5’TGGGTAACTTCGCTTT CGGG3’. Trizol reagent (Invitrogen, United States) was used for the extraction of RNA followed by DNases treatment. RevertAid First-strand cDNA synthesis kit (ThermoFisher Scientific, EU, Luthiana) was used for cDNA synthesis. A comparative threshold cycle value was used to determine the relative expression of the gene. Actin gene (AB181991.1) with primer pair 5’AA CTGGGATGACATGGGGAA3’ and 5’TTTTCTCTCTGTTGG CCTTGGG3’ was used for normalization of transcript level.

### ^32^P_i_ Labeling and Heat and Osmotic Stress Treatment

Leaf discs of 0.5 cm in size were taken from the center of collected leaf samples with the help of a vertical leaf disc puncher (Supplementary Figure 1). Two leaf discs for every replicate were taken. Leaf discs were metabolically labeled using labeling buffer 200 μl (MES-KOH 2.5 mM, pH 5.8, KCl 1 mM) containing carrier-free PO_4_^–3^ (5–10 μCi) in 2 ml Eppendorf tubes for overnight incubation, as described by Munnik et al. (1998) and Darwish et al. (2009). For PLD activity assay, n-butanol (0.5% v/v) was used as transphosphatidylation substrate (Darwish et al., 2009).

#### Heat Treatment

After overnight incubation for ^32^P_i_ labeling, samples were subjected to heat stress at 40°C using a heat block for the mentioned period of time, that is, 0, 7.5, 15, 30, and 60 min.

#### Osmotic Stress Treatment

For osmotic stress, 3–4-week-old leaf samples were treated with/without sorbitol by adding 200 μl of sorbitol in MES labeling buffer for 30 min and at 0, 600, and 1200 mM concentrations.

### Lipid Extraction and Analysis

Treatments were stopped by adding PCA (Perchloric acid) to the Eppendorf tubes and centrifuged at 13,000 rpm for 30 s. Leaving behind the leaf tissues in the tube, all the remaining material was discarded carefully, then 400 μl CMH [CHCL_3_/MeOH/HCl (50:100:1, by volume)] was added in the same tube and shook them for 5 min (until tissues turned colorless). By adding 400 μl of CHCL_3_ and 200 μl of NaCl (0.9% w/v), two-phase system was induced followed by 2 min centrifugation at 13,000 rpm. The rest of the lipid extraction and isolation was carried out by Munnik and Zarza (2013). Heat-activated K-oxalate (KOX^–^) impregnated TLC plates, using an alkaline solvent containing CHCL_3_, MeOH, 25% NH_3_ and H_2_O [90:70:4:16] constituents or an ethyl acetate system containing: EtAc/iso-octane/HCOOH/H2O (12:2:3:10, by vol.) were used to separate radioactive lipids (Munnik et al., 1998). Radioactively labeled phospholipids were visualized on an autoradiograph by overnight exposure of TLC plate to autoradiography film and quantified by using phosphoimaging (Typhon FLA 7000, GE Healthcare).

### Performance of Transgenic Lines of Wheat Under Heat and Combination of Stresses

Wild-type and transgenic lines (*AtPLC5OE*) of wheat were grown in pots under optimum conditions. These plants were subjected to heat stress (40°C) and drought together with heat stress (500 ml H_2_O + 40°C) in combination at the anthesis stage for 3 h daily for 14 days. Wild-type and transgenic lines of wheat were also grown at optimum temperature (25°C) as a control. Stay green character was recorded based on visual observation and leaf greenness. Data were recorded and analyzed in percentages.

## Results

### Expression of *AtPLC5* in Wheat Under Heat Stress

**FIGURE 1:**
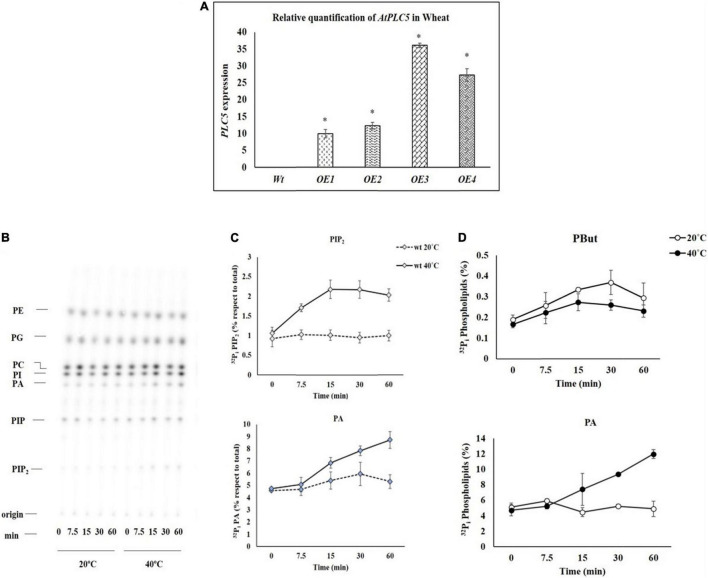
**(A)** The relative expression of *AtPLC5* transgenes in wheat normalized with actin gene determined through Q-PCR with a significant level *P* < 0.05 (*). **(B)** Heat stress excitation of PIP_2_ and PA formation in a time-dependent manner. Wheat leaf discs labeled with ^32^P_i_ and tested at 20°C (control) and 40°C (heat stress) for 0, 7.5, 15, 30, and 60 min time periods. An autoradiograph of a time-course experiment with two different temperatures, each lane representing extract (1/5th) of two leaf discs of two different leaves. **(C)** Quantification of PIP_2_ and PA, respectively, after heat treatment for said time. The experiment was independently repeated two times and similar results were obtained. **(D)** PLD activity. Pre-labeled wheat leaf discs were subjected to heat stress for said time periods in the presence of n-butanol (0.5% v/v). Lipids were extracted and separated by EtAc TLC. Quantification of PBut and PA levels was done by phosphoimaging. Abbreviations: PIP_2_, Phosphatidylinositol 4,5-bisphosphate; PIP, Phosphatidylinositol phosphate; PA, Phosphatidic acid; PI, Phosphatidylinositol; PC, Phosphatidylcholine; PE, Phosphatidylethanolamine; PG, Phosphatidylglycerol; CL, Cardiolipin.

The expression level of wild-type and *AtPLC5 OE* lines of wheat were determined by Q-PCR, relative to the expression of actin gene. For this, wild-type and transgenic lines (*AtPLC5 OE*) of wheat were subjected to heat stress at 40°C for 3 h at the anthesis stage. Leaf samples were collected immediately and stored in liquid nitrogen for further processing. Little to no expression was observed in *Wt.* Relative expression of *AtPLC5* shows a significant increase in all four over-expression transgenic lines of wheat (Figure 1A). The transgenic lines *OE1* and *OE2* showed 9.9X and 12.3X, while *OE3* and *OE4* lines showed 36.1X and 27.3X significant increase in the expression levels in comparison to the wild-type.

### Heat Stress Rapidly Stimulates Phosphatidylinositol 4,5-bisphosphate and Phosphatidic Acid Accumulation

To study the effect of the heat stress in wheat, the leaf disc of *Wt* was labeled with ^32^P isotope by keeping the leaf discs for overnight incubation in MES buffer and exposed to 20°C and 40°C by using heat block for 0, 7.5, 15, 30, and 60 min. Then, Perchloric acid (2.4% final concentration) was added to stop the reaction and crude lipids were extracted. Alkaline TLC (thin layer chromatography) plates were used to separate the lipids that were further quantified by phosphoimaging.

To investigate how fast the PIP_2_ and PA start to produce when subjected to heat stress, leaf discs of 4-week-old seedlings of wheat were exposed to heat stress for different time durations. The results of the time course experiment are presented in Figure 1B. The PIP_2_ and PA responses increased with the increase in duration of exposure to heat stress in a time-dependent manner (Mishkind et al., 2009), expression of PIP_2_ increased up to 2.2-fold, and PA increased up to 1.6-fold (Figure 1C) depending on the time of exposure.

#### Assay for Phospholipase D Activity

An experiment was carried out to investigate the distinct route of heat-induced PA generation. Either it occurs through PLC which cleaves PIP_2_ into IP3 and DAG that are further phosphorylated by DGK enzyme to generate PA or PA generation directly through PLD. Therefore, transphosphatidylation activity of PLD was employed. For this, pre-labeled leaf discs were subjected to heat stress (20°C and 40°C) at said time intervals in the presence of n-butanol (0.5% v/v). Ethyl acetate TLC was used to separate lipids and to track PLD-catalyzed phosphatidyl butanol (PBut) formation by phosphoimaging. Under these conditions, a small increase was observed in the PBut level at some time points, while a decrease in PA level was observed. In contrast, a simultaneous decrease in the accumulation of PBut level was observed during subsequent incubation at 40°C with an increase in the level of PA (Figure 1D).

### Mature Leaves Accumulate More Phosphatidylinositol 4,5-bisphosphate

**FIGURE 2:**
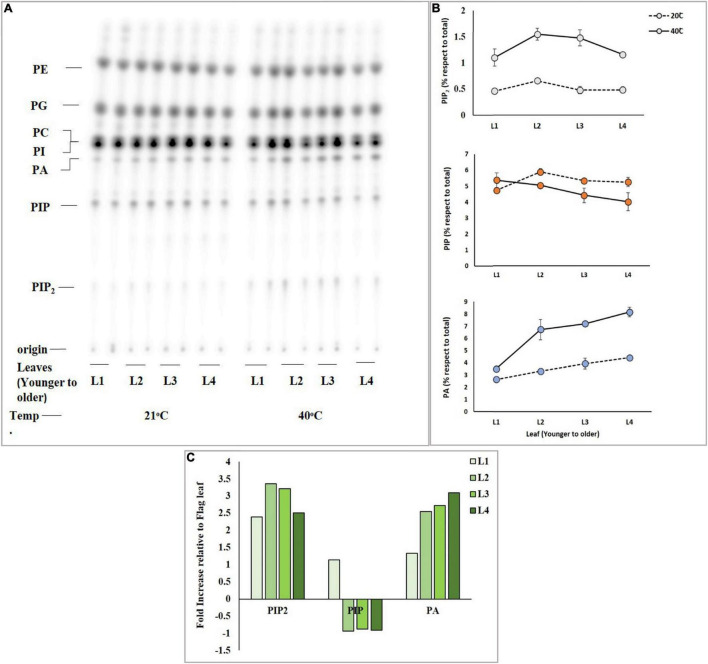
High temperature triggers the rapid accumulation of PIP_2_ and PA in mature leaves. Wheat leaf discs were pre-labeled overnight with ^32^PO_4_^3–^, incubated for 15 min at 21°C and 40°C, and lipid extraction and separation were carried out by using alkaline TLC. Panel **(A)** shows autoradiograph of lipid TLC. **(B)** PIP_2_, PIP, and PA levels were quantified by densitometry of autoradiograph shown in panel **(A)**. **(C)** Summary of fold increase in levels of PIP_2_, PIP, and PA relative to *t* = 0. The experiment was repeated three times independently and similar results were obtained. See Figure 1 for the definition of abbreviation.

Differential response of leaves of the same tiller of the same wheat plant was analyzed for accumulation of PIP_2_ upon exposure to heat stress. An experiment was designed to investigate which leaf (either younger or mature leaves) responds more efficiently to heat stress by producing a sufficient amount of PIP_2_, PIP, and PA, and four different leaves including the newly emerged leaf of the same tiller of Faisalabad-2008 wheat cultivar were taken and labeled radioactively by overnight incubation. ^32^P_i_ labeled leaf discs of four different leaves were subjected to heat stress at 21°C and 40°C for 15 min (Figure 2A). The results demonstrated a considerable gradual increase in PIP_2_ and PA levels among leaves with the increase in temperature, while the level of PIP declined (Figure 2B) upon receiving heat stress as described previously (Mishkind et al., 2009). The mature leaves showed a 3.3-fold increase in PIP_2_ and a 2.6-fold in PA but a 1-fold gradual decrease in PIP production was observed as compared to younger leaves (Figure 2C; Wang X. et al., 2021).

### Phosphatidylinositol 4,5-bisphosphate Level Increases at Anthesis Stage in Response to Heat Stress

**FIGURE 3:**
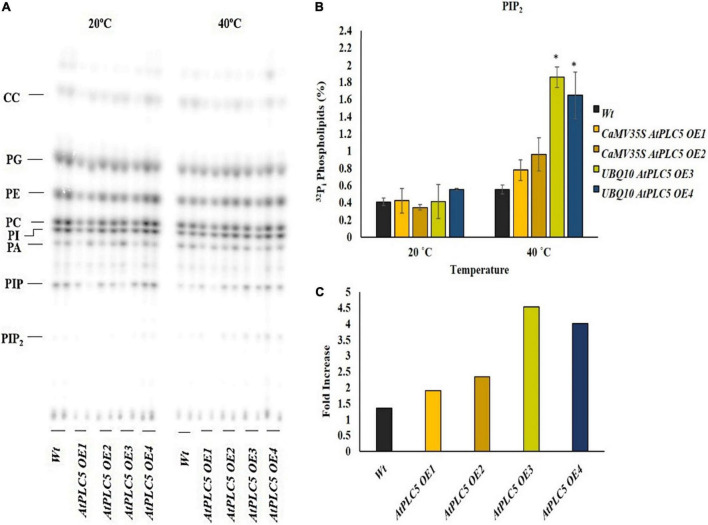
Heat stress increases the production of PIP_2_ at the anthesis stage. A small section (∼0.5 cm) of leaf disc of transgenic wheat lines and wild-type were taken at the anthesis stage, radioactively labeled O/N with ^32^P_i_, and incubated for 30 min at 20°C and 40°C using a heat block. **(A)** An autoradiograph of alkaline TLC showing a complete lipid profile. **(B)** Quantified level of PIP_2_ after heat treatment at 20°C and 40°C. **(C)** Summary of fold increase transgenic lines show with respect to wild-type. Student *t*-test was used to determine significant differences between wild-type and transgenic lines of *AtPLC5* at a significance level of *P* < 0.05 (*).

The wheat anthesis stage is very sensitive to high temperatures. A rise in temperature beyond 25°C drastically affects pollen viability, decreases the chances of seed setting, and results in lesser crop yield. The lipid profile of transgenic wheat plants containing two different promoters and their response to heat stress at the anthesis stage was determined by subjecting their labeled leaf discs to 40°C for 30 min (Figure 3A). The lipid profile patterns showed a rise in PIP_2_ levels in response to heat stress in transgenic and wild-type wheat plants (Figure 3B). The PIP_2_ level revealed a significant increase in the transgenic lines under different promotors in comparison to the wild-type. While the wild-type showed little to no increase, the transgenic lines, *OE1* and *OE2*, depicted a 2.0- to 2.5-fold increase, whereas, *OE3* and *OE4* transgenic plants showed ∼4.5-fold and 4-fold increase in PIP_2_ production, respectively (Figure 3C).

### Osmotic Stress Triggers the Phosphatidylinositol 4,5-bisphosphate Production in *AtPLC5* Over-Expressing Wheat Lines

**FIGURE 4:**
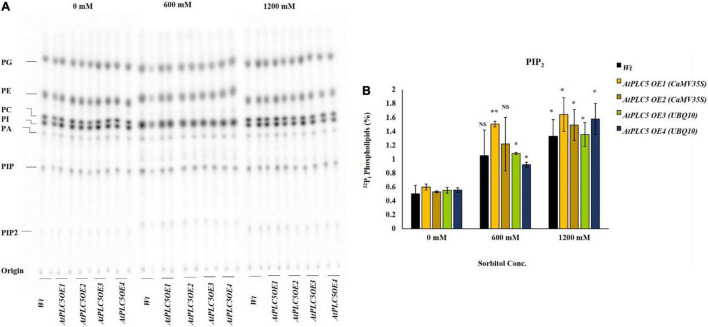
PIP_2_ accumulation in wheat *AtPLC5 OE* lines at different levels of osmotic stress. Overnight labeled leaf disc treated with different concentrations of sorbitol (0 mM, 600 mM, 1200 mM) dissolve in MES buffer for 30 min. Lipids were extracted, separated, and quantified by phosphoimaging. **(A)** A typical autoradiograph of structural lipids with each lane containing lipid extract of two leaf discs. **(B)** ^32^P_i_ level of PIP_2_ of the wild-type and *AtPLC5 OE* transgenic lines of wheat under control conditions and at said sorbitol concentrations. Data represented as means ± SD (*n* = 2). Two independent experiments were carried out with similar results obtained. Statistically significant differences at *P* < 0.05 (*) and at *P* < 0.01 (**) were observed in *AtPLC5 OE* of wheat at different sorbitol concentrations, based on the student’s *t*-test.

The role of osmotic stress in the production of lipid was analyzed in *Wt* and *AtPLC5* over-expressing wheat lines. ^32^P_i_ labeling of 4-week-old plant leaf discs was performed to test various concentrations of sorbitol to mimic water stress. Leaf discs were treated with 0, 600, and 1200 mM sorbitol pre-dissolved in MES labeling buffer for 30 min before extraction. Five percent perchloric acid (PCA) was used to stop preceding the reaction further and crude lipids were extracted. Potassium oxalate (KOX^–^)-treated TLC plates were used to separate the lipids and phosphoimaged for quantification purposes (Figure 4A).

Under control conditions, the amount of PIP_2_ remained the same among *AtPLC5 OE* lines and wild-type (Figure 4B). A relative significant [*P* < 0.05 (*), *P* < 0.01 (**)] increase in PIP_2_ level was observed in *AtPLC5 OE* lines (*OE*1, *OE2*, *OE3*, and *OE4*) under different promoters at 600 mM sorbitol concentration, while a non-significant increase was observed in wild-type. Upon sorbitol treatment of 1200 mM, a significant increase in PIP_2_ level was observed in wild-type (∼2.7-fold) and *AtPLC5* over-expression lines (∼3.3-fold) as compared to control condition, whereas non-significant differences were observed between the wild-type and *AtPLC5-OE* lines at 600 mM and 1200 mM sorbitol concentrations. However, the *AtPLC5 OE4* line showed a significant (*P* < 0.05) increase (∼1.8 and ∼3.2-fold) in the PIP_2_ level at 600 mM and 1200 mM sorbitol treatment, respectively. The PA and PIP responses in wild-type and *AtPLC5 OE* lines appeared to be almost similar (a slight increase was observed in *AtPLC5* over-expression lines) at said levels of sorbitol concentrations.

### Combination of Heat and Osmotic Stress Elicit Phosphatidylinositol 4,5-bisphosphate Accumulation in *AtPLC5* Over-Expression Line

Usually, owing to the duration of the wheat cultivation, the crop faces several stresses at the same time. The occurrence of more than one stress in combination severely affects plant growth and development. Moreover, any visible symptom of heat and osmotic stress cannot be detected at the early stages of plant growth. To determine the response of *AtPLC5* in transgenic wheat under the combination of heat and osmotic stress conditions, 4-week-old plantlets were tested at 40°C and 600 mM sorbitol for 30 min simultaneously.

**FIGURE 5:**
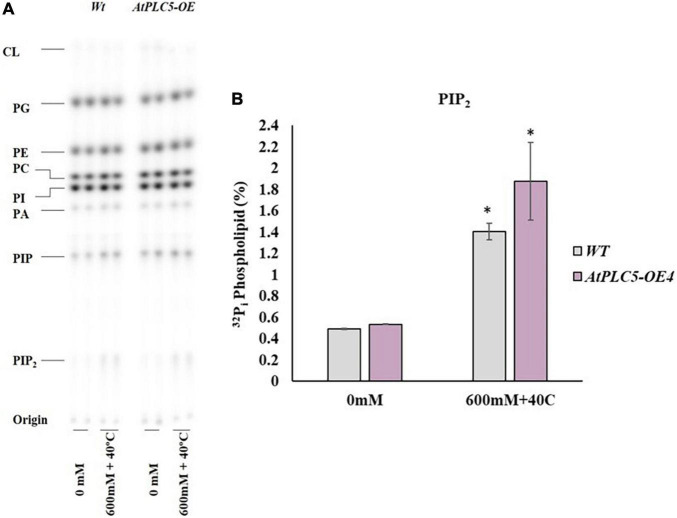
The occurrence of stresses in combination elicits PIP_2_ accumulation in *AtPLC5 OE4* lines in wheat. Radioactively labeled leaf discs were treated with 600 mM sorbitol at 40°C for 30 min. Lipids were extracted, separated by Thin Layer Chromatography plates, and quantified by phosphoimaging. **(A)** A typical autoradiograph of lipids with each lane containing 1/5th lipid extract of two leaf discs. **(B)** ^32^P_i_ level of PIP_2_ of the wild-type and *AtPLC5 OE4* line under control conditions and at co-stress of heat and osmotic stress situation. Data represented as means ± SD (*n* = 2). Two independent experiments were carried out with similar results. Statistically significant differences at *P* < 0.05 (*) were observed between the *AtPLC5 OE4* line of wheat and wild-types under said stress conditions, based on the student’s *t*-test.

The amount of PIP_2_ under control/non-treated conditions was observed (Figure 5A) to be the same among the *AtPLC5 OE4* line and wild-type (Figure 5B). A relative increase in PIP_2_ was observed at a significance level of *P* < 0.05 (*) in wild-type and *AtPLC5 OE* lines (containing *UBQ10* promoter) at 600 mM sorbitol concentration at 40°C temperature when compared to the control condition. Under co-stress conditions, a significant increase of 2.8-folds in PIP_2_ was observed in wild-type and 3.5-folds in *AtPLC5* over-expression line in a controlled environment.

### Performance of *AtPLC5* Overexpression Line Under Abiotic Stress

**FIGURE 6:**
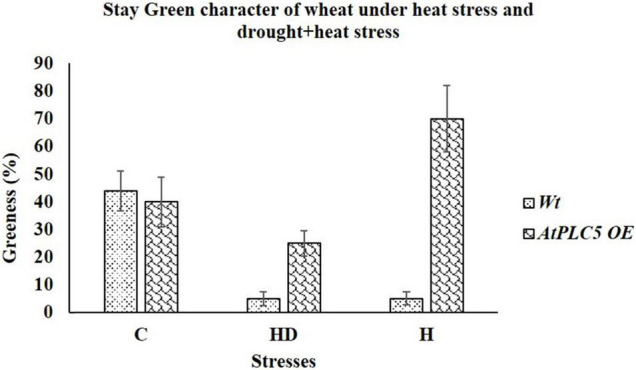
Morphological evaluation of *Wt* and *AtPLC5* overexpression lines of wheat under heat stress and under the combined effect of heat and drought stress. Stay green character was recorded based on visual observation and leaf greenness. Data (*n* = 26, ±SD) were recorded and analyzed in percentage.

To check the contribution of *AtPLC5* overexpression in wheat physiology or its agronomic performance, two different experimental conditions were set up. First, we tested the physical response of *AtPLC5 OE* lines under heat stress at 40°C and second, when stress was applied in combination, such as heat with drought stress (40°C + 500 ml H_2_O). After the treatment of 2 weeks, we observed the stay-green character in *Wt* and *AtPLC5* transgenics of wheat. We observed that at optimum conditions (32°C), *Wt* possesses ∼32%, while *AtPLC5* transgenics possess ∼40% greenness (Figure 6). When stress was applied in combination with heat (40°C) and drought (500 ml water), we observed visible leaf necrosis in *Wt* (∼5% greenness) and *AtPLC5* transgenic plants of wheat (∼25% greenness). Interestingly, we found that the transgenic plants of wheat that received treatment of heat stress (40°C) show the ∼70% stay-green character as compared to *Wt*.

## Discussion

Abiotic stresses can elicit a series of plant responses. Membrane plays an important role in vesicle transport and cell signaling not only through host-specific proteins but also provides a substrate for the production of lipid (as a second messenger). In addition to the role of lipids as components of membrane structure, they also work as a signal transducer, component of coordinated regulatory activator, and stimulate the expression of specialized proteins and trigger cellular responses to environmental cues (Hou et al., 2016; Kosová et al., 2018; Munnik et al., 2021). Phospholipases on the plasma membrane are the first receptors to receive environmental signals and respond accordingly. PLCs due to their regulatory roles in stress management have been extensively investigated in different plant species. It has been established that stress causes a synergistic increase in PIP_2_ levels and free calcium, which enhances IP_3_ synthesis and further releases cytosolic calcium through PI-PLC activity (Hunt et al., 2004; Gao et al., 2014; Zhang et al., 2014). Heat shock induces a rapid increase of Ca^+2^ in the cytoplasm, probably from intracellular reserves and extracellular sources. It is reported that Ca^+2^/calmodulin pathway is involved in thermotolerance. It is logical to claim that Ca^+2^ channels could be used as a thermosensor (Gao et al., 2012; Hayes et al., 2021). However, it is still a challenging task to identify the primary heat-activated Ca^+2^ channel.

Previously, PIP_2_ and PA abundance had been observed in *Arabidopsis* within 2 min of onset of heat (40°C) stress, and it was mediated by PLD and PIPK (Mishkind et al., 2009). In the current study, we investigated the stimulation of heat-induced PIP_2_ and PA accumulation in *Triticum aestivum* L. and observed that their induction proceeded in a time-dependent manner. The rapid rise in PIP_2_ level was evident with the onset of heat (40°C) that reached 2.2-folds in just 15 min and continue to increase with the increase in the duration of heat stress. However, after 60 min, the PIP_2_ level started to decline, which might indicate the stress-induced damage caused to the plasma membrane. In the current study, it was observed that the PA accumulation started just after 7.5 min of the onset of heat stress, and kept on increasing continuously with the increase in the duration of heat stress. The quick abundance of PIP_2_ and PA indicates the synthesis of these signaling lipids associated with thermosensing. Although it is still unclear how the elevated temperature activates these lipid-modifying enzymes, this increase in PIP_2_ and PA is either caused by PIP5K, PLC, or PLD activity, which is yet to be determined. It is reported previously that PA induction is closely associated with the activation of PLD under heat stress (Shiva et al., 2020; Hayes et al., 2021); however, it has been observed that in wheat at 40°C, PBut level seems to decrease while the total PBut content remains in lower limit. In contrast, PA level seems to increase in a time-dependent manner. It is still unknown which other factors are involved in the generation of PA through PLD or PLC. Similarly, it is yet to be explored what circumstances help in the activation/inhibition of PLD or PLC.

Plant leaves serve as a sensor for biotic and abiotic stresses. A slight change in the surrounding temperature is usually sensed by the plant through their leaves. The present study investigated PIP_2_ and PA responses in younger to older leaves against heat stress. We also observed PIP (Phosphatidylinositol monophosphate) response. Upon onset of heat stress (40°C), the young leaves depicted minor elevation in PIP_2_, PIP, and PA and contributed accordingly to stress responses as compared to mature leaves which showed a gradual increase up to 3.4-folds in PIP_2_ and PA accumulation, while illustrated 1-fold decrease in PIP level. Therefore, it could be suggested that although the younger leaves have actively dividing cells, they are quite sensitive to heat stress Zhang et al. (2014) reported a 16-fold increase in *TaPLC1* expression level in older leaves upon salt and drought stress. This could be implied that an increase in expression in response to environmental changes might be considered an adaptive mechanism to manage abiotic stresses.

In the current study, PIP_2_ response was observed to be similar in wild-type (Faisalabad-2008) and *AtPLC5* over-expressing lines of wheat under normal conditions (20°C). However, heat stress (40°C) at the anthesis stage caused a stronger and significant rise in PIP_2_ level in *AtPLC5* over-expression lines (Figure 3B) as compared to wild-type that ultimately helped the plant to adapt/tolerate fluctuations in temperature and grain formation sustaining the crop yield. We also compared the strength of two constitutive promoters (*CaMV35S* and *UBQ10*). *UBQ10* promoter indicated relatively higher expression of *AtPLC5* in *OE3* and *OE4* lines with a consequent significant increase of ∼4.5-folds in PIP_2_ accumulation as compared to *AtPLC5* expression driven under *CaMV35S* promoter in *OE1* and *OE2* transgenic wheat. Zhang et al. (2018c) reported a 12-fold increase in PIP_2_ level at the onset of osmotic stress in *PLC5OE* lines containing *UBQ10* promoter in 6-day-old seedlings of *Arabidopsis thaliana* (Zhang et al., 2018c), which is in agreement with our findings and increase in PLC activity.

PI-PLC as a stress mediator had been reported along with their isoforms in many plants including maize (Apostolakos et al., 2008), rice (Darwish et al., 2009; Singh et al., 2013), tobacco (Helling et al., 2006), tomato (Vossen et al., 2010), cotton (Zhang et al., 2018a), soybean (Wang F. et al., 2015), brassica (Das et al., 2005), *Arabidopsis* (Gao et al., 2014), and wheat (Wang X. et al., 2021). Recent findings illustrated the over-expression of *TaPLC1* aided in improved salt, drought, heat, and cold stress tolerance in wheat (Khalil et al., 2011; Wang Y. et al., 2020; Wang X. et al., 2021). PIP_2_, as a PLC substrate is hardly detected in plants’ plasma membrane under normal conditions, while its level significantly increased under osmotic stress, for example, cold, salinity, or heat stress (Darwish et al., 2009; Mishkind et al., 2009; Arisz et al., 2013; Munnik, 2014; Zhang et al., 2014). In the present study, it was observed that the lines that showed more PIP_2_ accumulation also revealed more transcript levels through real-time quantitative PCR. In addition, we also observed that these lines retained their stay green character relatively for a longer period of time when exposed continuously for 14 days to heat stress.

The structural lipids like PC (Phosphatidylcholine), PG (Phosphatidylglycerol), and PA (Phosphatidic acid) at the anthesis stage of wheat were reported to drop under high temperatures (Narayanan et al., 2016; Djanaguiraman et al., 2020). Likewise, we also observed a slight decrease in PA in our *AtPLC5* over-expression lines of wheat during anthesis at 40°C. However, ∼2.2-fold increase in PA accumulation was observed in the wild when subjected to heat stress (40°C for 30 min). This increase might reflect the activity of PLD as previously reported by Hayes et al. (2021).

Upon rising environmental temperature, plants with sufficient water resources transpire more rapidly to keep their leaves cool, while on water scarcity in hot conditions, leaves close their stomata to prevent water loss through evaporation and to maintain their cells membrane integrity. Lee et al. (2007) reported PIP_2_ to be an important precursor for stomatal opening, as detected previously in the closed stomata phenotype of the *PLC5OE* line in *Arabidopsis.* In this study, sorbitol was used to mimic drought/osmotic stress in wheat and to observe its effect on the PIP_2_ level. Interestingly, a significant increase in PIP_2_ level was observed upon osmotic stress in *AtPLC5* overexpression lines of wheat, this might result in the enhanced hydrolytic activity of *PLC5* which might lead to an increase in PIP_2_ hydrolysis resulting in a subsequent increase in IP_3_ that might further be metabolized into IP_6_ which facilitate the stomatal closure by activating the release of Ca^+2^ from intracellular channels (Zhang et al., 2018b,c). In addition, in the current study, we observed that wheat transgenic lines containing *CaMV35S* promoter induced significantly higher PIP_2_ that matched with findings previously reported by Zhai et al. (2013). It is explained that *ZmPI-PLC1* expressed under *CaMV35S* promoter induced drought tolerance in transgenic tobacco (Ruelland et al., 2015).

However, a detailed phosphoimager-based densitometry study demonstrated a meager decrease in PI and PE levels and a slight increase in PC and PG levels, when exposed to osmotic stresses. An increase in the level of cardiolipin (CL) was observed in *OE* lines of transgenic wheat. In plants, PG (phosphatidylglycerol) was found to be mainly present in the thylakoid membrane of chloroplast and supposed to be involved in the photosynthetic electron transport chain (Hagio et al., 2002; Kobayashi et al., 2017). Previous reports have suggested the prerequisite presence of PG for chloroplast biogenesis, as its deficiency yielded a pale-yellow green phenotype, indicating the failure of establishing thylakoid membrane networks inside leaf chloroplast (Haselier et al., 2010; Kobayashi et al., 2015). Interestingly, an increase in PG level of overexpressor lines of wheat was observed, which means they remained photosynthetically active when exposed to abiotic stress and could accumulate more synthates, more synthates mean more nutrients available to be assimilated during grain filling leading to enhanced crop productivity, which might ultimately yield higher grain and biomass.

Phosphatidylinositol 4,5-bisphosphate is claimed to be a PLC substrate in animals, its concentration is relatively hard to detect in the plasma membrane of plants where PLC activity mostly resides (Van Leeuwen et al., 2007; Munnik, 2014). In contrast to PIP_2_, PI4P is 20–30 times more abundant in plasma membrane under normal conditions. Under stress conditions such as abscisic acid (ABA), salinity, heat, or hyperosmotic stress, the level of PIP_2_ increased (Darwish et al., 2009; Mishkind et al., 2009; Zhang et al., 2018b), while the level of PI4P has been reported to drop in response to these stresses (Arisz et al., 2013). But does it go down due to conversion into PIP_2_ or PIP is an assumed substrate of PLC in the plant? Also, it remained debatable, whether this reflected the hydrolysis by phosphatase or a PLC or is a result of PIP5K activation. Further research is needed to decipher the exact role of PLC in wheat and the downstream process of PA, PPIs, and IPPs production and accumulation.

## Data Availability Statement

The original contributions presented in the study are included in the article/Supplementary Material, further inquiries can be directed to the corresponding author.

## Author Contributions

NA, MA, and NS conceived and designed the research. NA conducted the research experiments. NA and KI evaluated the data. MA, NS, and MT provided the research material. NA and MA wrote the manuscript. SM, MT, and NS critically reviewed and edited the manuscript. All authors contributed to the article and approved the submitted version.

## Conflict of Interest

The authors declare that the research was conducted in the absence of any commercial or financial relationships that could be construed as a potential conflict of interest.

## Publisher’s Note

All claims expressed in this article are solely those of the authors and do not necessarily represent those of their affiliated organizations, or those of the publisher, the editors and the reviewers. Any product that may be evaluated in this article, or claim that may be made by its manufacturer, is not guaranteed or endorsed by the publisher.

## Abbreviations

DAG: diacylglycerol
DGK: diacylglycerol kinase
IP_3_: inositol 1,4,5 trisphosphate
IPP: inositol polyphosphate
OE: overexpression
PA: phosphatidic acid
PIP: phosphatidylinositol monophosphate
PIP_2_: phosphatidylinositol 4,5-bisphosphate
PIPK: phosphatidylinositol phosphate kinase
PI-PLC: phosphoinositide specific phospholipase C
PLD: phospholipase D.
