# Supplementary figures and images for: 32Pi Labeled Transgenic Wheat Shows the Accumulation of Phosphatidylinositol 4,5-bisphosphate and Phosphatidic Acid Under Heat and Osmotic Stress
Source: Front Plant Sci. 2022 Jun 14;13:881188. doi: 10.3389/fpls.2022.881188 (PMC9237509; doi:10.3389/fpls.2022.881188)

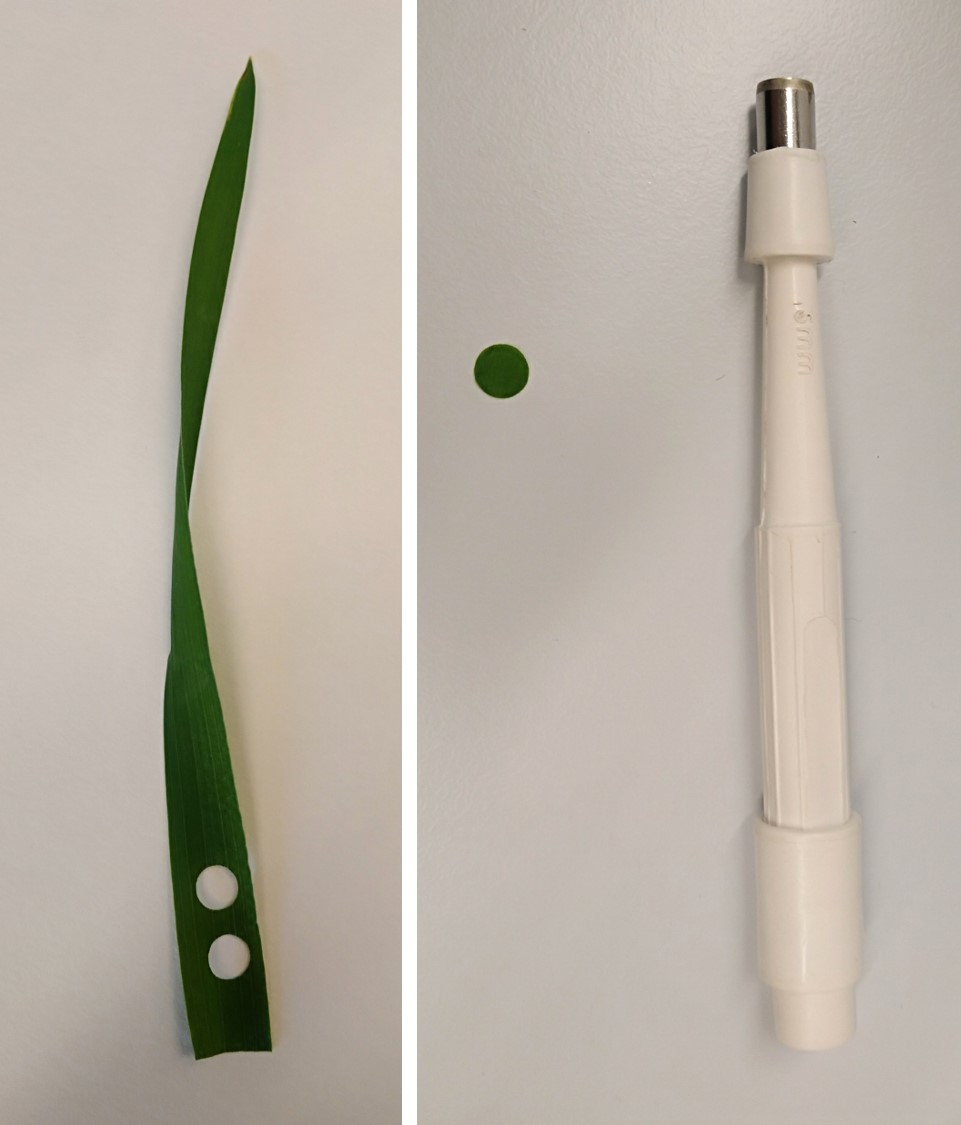

Supplement: Supplementary file 1 [file Image_1.JPEG]
